# Novel Molecular Targets for Chemoprevention in Malignancies of the Head and Neck

**DOI:** 10.3390/cancers9090113

**Published:** 2017-08-31

**Authors:** Aarti Bhatia, Barbara Burtness

**Affiliations:** Yale Comprehensive Cancer Center, Yale University School of Medicine, New Haven, CT 06520, USA; barbara.burtness@yale.edu

**Keywords:** head and neck cancer, oral cancer, clinical trial, second primary tumor, chemoprevention, retinoids

## Abstract

Cancers of the head and neck region are among the leading causes of cancer-related mortalities worldwide. Oral leukoplakia and erythroplakia are identified as precursor lesions to malignancy. Patients cured of an initial primary head and neck cancer are also susceptible to developing second primary tumors due to cancerization of their mucosal field. Multi-step acquisition of genetic mutations leading to tumorigenesis and development of invasive cancer has been previously described. Recently, whole exome sequencing of tumor specimens has helped to identify driver mutations in this disease. For these reasons, chemoprevention or the use of systemic or biologic agents to prevent carcinogenesis is an attractive concept in head and neck cancers. Nonetheless, despite extensive clinical research in this field over the past couple decades, no standard of care option has emerged. This review article reports on targeted interventions that have been attempted in clinical trials to date, and focuses on novel molecular pathways and drugs in development that are worthy of being tested for this indication as part of future endeavors.

## 1. Introduction

Over 60,000 new cases of squamous cell cancers of the head and neck (SCCHN) will be diagnosed in the United States alone in 2017, and over 13,000 people are expected to die from their disease [1]. Leukoplakia and erythroplakia are both recognized as oral premalignant lesions (OPML) and are known to precede invasive oral carcinoma by months or years [2,3,4,5,6,7]. However, the risk of malignant transformation is known to be highly variable among populations [6,8,9]. The majority of SCCHN could be caused by environmental exposure to tobacco and alcohol [10]. This is believed to be a key trigger for the development of OPML and their eventual transformation into invasive cancers. Increasingly over the past couple of decades, we are also witnessing a rising incidence of human papillomavirus (HPV)-associated oropharynx cancers, particularly among younger men in the economically developed world [11]. Despite recent advances in multi-disciplinary treatment approaches including surgery, radiation, and chemotherapy, the five-year overall survival (OS) for patients with SCCHN is only about 40–60%. And often times, survivors live with long-term functional impairment (xerostomia, dysphagia, feeding tube dependence) from treatment-related morbidity. Patients cured from an initial cancer are also at significantly increased risk of developing a second primary tumor (SPT) [12,13]. A combined analysis of 13 international cancer registries showed that the cumulative risk of a SPT over 20 years reaches between 30–40% with no plateauing over time, and that these most commonly develop in the aero-digestive tract [12]. SPT therefore, are a major cause of increased morbidity and mortality among survivors of an initial SCCHN primary [14,15,16].

## 2. Rationale and Defining Populations at Risk

For these reasons, chemoprevention has long been thought of as an attractive concept in SCCHN. The term chemoprevention was first introduced by Michael Sporn and his colleagues in 1976, and was defined as the use of natural or synthetic chemicals for the reversal, suppression, or prevention of invasive carcinoma [17]. Two important hypotheses highlight the need for SCCHN chemoprevention. The first is the field cancerization theory, which was proposed by Danely Slaughter and colleagues in 1953, and refers to the effect on the upper aero-digestive tract mucosa of the chronic exposure to environmental carcinogens (tobacco and alcohol) [18]. He observed that clinically normal appearing oral mucosa adjacent to resected malignant lesions exhibited microscopic changes of pre-malignancy. The entire mucosal field was therefore thought to be condemned for progressive carcinogenesis. Califano et al then proposed the genetic progression model for head and neck cancers [19]. They examined 10 tumor suppressor loci in oral lesions categorized as hyperplasia, dysplasia, carcinoma in situ, and invasive oral cancer, and found that progression from precancerous to cancerous lesions was a multi-step process of oncogenic activation and silencing of tumor suppressor genes. There is a general order of acquisition of genetic changes, which cumulatively leads to malignant transformation. More recently, investigators have reported that most genetic changes occur prior to carcinogenesis, and that gene expression profiles differ between normal mucosa, OPML, and invasive cancer [20]. Loss of heterozygosity (LOH) at 3p14 (containing the tumor suppressor gene *FHIT*) and 9p21 (containing the cell cycle regulating gene p16), and augmentation of 11q13 (which houses cyclin D1) are now recognized as early events in tumorigenesis [21]. TP53 mutation and proliferative signaling through the epidermal growth factor receptor (EGFR) and cyclooxygenase-2 (COX-2) pathways are later events in this process [22,23]. As a correlative study component of the Phase III Erlotinib Prevention of Oral Cancer (EPOC) trial, investigators validated the use of LOH at high-risk loci as portending increased oral cancer risk among patients with OPML [24]. Not unexpectedly, it also correlated with increased EGFR gene copy number.

Given that the genetic progression of events in head and neck carcinogenesis is being elucidated, it could theoretically provide a framework to design future studies of SCCHN chemoprevention using novel agents that target known drivers of carcinogenesis. Three discrete populations could potentially be candidate for these trials: (1) individuals with excessive exposure to tobacco or alcohol who are at high risk for developing SCCHN; (2) individuals with OPML who harbor high-risk features of their lesions evolving into invasive cancers; and (3) patients who have been cured of an initial SCCHN primary, but remain at risk of developing SPT.

## 3. Methods

This review was based on a comprehensive search on PubMed, Google Scholar and Medline Ovid using medical subject heading (MeSH) terms “head and neck cancer chemoprevention”, “cancer chemoprevention”, and “head and neck cancer chemoprevention trials”. Inclusion criteria were studies published in humans, as well as published preclinical data in human cell lines and animal models. Patient information, including treatment response, recurrence, and follow-up time, when available, was extracted. Information presented in Table 3 was obtained from clinicaltrials.gov using the same search terms.

## 4. Past Chemoprevention Efforts

### 4.1. Retinoids

Early observational studies demonstrated that vitamin A deficiency in animal and human systems led to abnormal differentiation of epithelial cells and metaplasia, which could be reversed by retinoids [25,26]. Retinoids are postulated to work by restoring nuclear retinoid receptor beta (RAR beta) mRNA expression, which resets an abnormally proliferating clone of premalignant cells into normal growth and differentiation [27]. Initial chemoprevention efforts therefore focused on naturally occurring and synthetic vitamin A analogues (vitamin A, beta-carotene, cis-retinoic acid, etretinate, and retinyl palmitate). Hong et al reported that 1–2 mg/kg daily dose of 13-cisretinoic acid (13-CRA), given to patients with oral leukoplakia significantly decreased the size of the lesions and completely reversed dysplasia in 54% of patients, compared with a placebo [28]. Relapse occurred in over half of the responders 2–3 months after drug cessation. They then randomized 103 patients curatively treated for stages I-IV SCCHN, to daily high-dose 13-CRA (50–100 mg/m^2^ body surface area (BSA)) vs. placebo for 12 months [29]. Although there were no significant differences in local or distant recurrence, the group receiving the study drug had significantly fewer SPT (4% vs. 24%, *p* = 0.005). However, 13-CRA did not prolong OS, with the majority of patients being alive in both study arms [30]. In the landmark Phase III trial of retinoid intervention, patients curatively treated for Stage I–II SCCHN were then randomized for 30 mg daily dose of 13-CRA or a placebo for three years [31]. A lower dose was chosen because of the observed high toxicity rate and low compliance to treatment with the previously tested high doses of 13-CRA. Patients were monitored for four years beyond treatment completion, and there were no significant differences in SPT or OS between the two study arms. Current smoking was found to be associated with the acquisition of SPT and with decreased OS. The exclusion of advanced stage patients, the substantially lower dose of retinoid used in this study, and the possible effect of smoking cessation on SPT and OS made it difficult to confirm or refute the beneficial chemoprevention findings from earlier studies. Currently, no sound evidence exists to support its clinical use.

### 4.2. Cyclooxygenase-2 (COX-2) Inhibitors

COX-2 plays a role in the progression of epithelial tumors by influencing cellular functions such as apoptosis, angiogenesis, proliferation, invasion, and metastasis [32]. Renkonen et al. found an increasing gradient of cyclo-oxygenase-2 (Cox-2) expression when studied by immunohistochemistry in normal oral mucosa, dysplastic epithelium, and invasive squamous cell carcinoma (SCC) [33]. Shiotani et al. reported that although COX-2 protein was barely expressed in normal rat tongue epithelia on Western Blot analysis, there was a six-fold increased expression in 4-nitroquinolone-1-oxide (4-NQO)-induced squamous cell carcinoma (SCC) lesions [34]. Wang et al. first reported on the chemopreventive efficacy of celecoxib in oral cancer development in a nude mouse model [35]. The mice were intradermally inoculated with oral carcinoma cells, and then fed a celecoxib-supplemented diet or regular diet. Celecoxib significantly deferred tumor cell growth and decreased tumor volume. There was also decreased neo-vascularization in the tumor sites, suggesting an anti-angiogenic effect. Saba et al. studied COX-2 expression by immunohistochemistry in non-cancerous tissue, progressive stages of OPML and carcinoma in situ (CIS) lesions from non-smoking, non-cancer subjects [36]. They found that COX-2 expression incrementally increased through premalignancy and was less intense in the severe dysplasia/CIS stage as well as in malignant cells. Cumulative data provided the rationale for studying COX-2 inhibitors for their chemopreventive efficacy in OPML. However, results from observational and case-control studies are conflicting. A questionnaire-based matched case-control study of 529 patients with SCCHN found that aspirin use decreased the risk of SCCHN by 25%, and the benefit accumulated with length of treatment [37]. Women derived greater benefit, while patients with a history of heavy tobacco and alcohol consumption did not. In a blind, randomized trial of oral ketorolac rinse solution, there was no difference in the extent of leukoplakia versus placebo [38]. Wirth et al. measured changes in prostaglandin E-2 (PGE-2) levels in 22 patients with OPML treated with celecoxib [39]. In 18 pairs of biopsies from baseline and 12-weeks post-treatment, PGE-2 levels decreased by 38% (*p* = 0.002). In 12 biopsies (67%, *p* = 0.0129) severity of dysplasia improved after 12 weeks, and in 8 of 11 biopsies (73%, *p* = 0.0703), there was continued improvement beyond 12 months. COX-2 inhibitor use was found to significantly accentuate the risk of cardiac complications in placebo-controlled trials for the prevention of colorectal adenomas [40,41], leading to an eventual decline in their use. Also, a recently published meta-analysis of eleven observational studies suggested that overall, the use of non-steroidal anti-inflammatory drugs (NSAID) does not significantly decrease the risk of SCCHN (OR = 0.95; 95% confidence interval (CI), 0.81–1.11) [42]. Based on these data, they currently have no established role in head and neck cancer chemoprevention.

### 4.3. EGFR Inhibition

The epidermal growth factor receptor (EGFR)-signal transducer and activator of transcription (STAT)-3 signaling pathway plays a key role in SCCHN growth, survival, and prognosis [43]. Shin et al. demonstrated significant upregulation in EGFR expression between dysplastic tissue and SCCHN [44]. Leeman-Neill et al. reported on a 4-NQO-induced murine model of oral carcinogenesis which was utilized to investigate the chemopreventive activities of erlotinib and guggulipid (STAT-3 inhibiting compound) [43]. Dietary guggulipid did not protect against oral carcinogenesis. However, mice on an erlotinib-supplemented diet demonstrated a 69% decrease (*p* < 0.001) in the development of premalignant and malignant lesions versus those on a control diet. With the rationale to test EGFR inhibitors in the clinical setting, a phase II trial of cetuximab, a monoclonal anti-EGFR antibody, in high-risk pre-cancerous lesions of the upper aerodigestive tract, demonstrated complete reversal of dysplasia in four of 12 patients (33%) treated with weekly doses for eight weeks versus zero of five patients in the observation arm [45]. However, intravenous treatment with cetuximab posed a limitation to the longer duration of testing. The convenience and availability of oral EGFR tyrosine kinase inhibitors (TKI) led to the design and implementation of the phase III randomized Erlotinib prevention of oral cancer (EPOC) trial [24]. 150 patients, who were determined to have high-risk OPML based on specific LOH profiles and previous history of oral cancer, were randomized to treatment with oral erlotinib 150 mg daily dose or placebo. Three-year oral cancer-free survival (CFS) was lower for the high-risk LOH group versus the low-risk LOH group (74% vs. 87%, HR, 2.19; 95% CI, 1.25–3.83; *p* = 0.01). Although an increase in EGFR gene copy number was associated with high-risk LOH profile (*p* < 0.001) and lower CFS (*p* = 0.01), it did not portend greater efficacy with erlotinib. There was also no significant difference in three-year CFS between arms [hazard ratio (HR), 1.27; 95% CI, 0.68–2.38; *p* = 0.45]. These results do not support use of erlotinib in the chemoprevention setting.

A combination of EGFR TKI and celecoxib was studied for its effects on SCCHN cell lines and showed significant G1 arrest, apoptosis, and suppressed capillary formation of endothelium, leading to inhibited growth of all five cell lines tested, and suggesting there might be an additive or synergistic decrease in COX-2 expression [46]. Zhang et al. then reported effects of celecoxib alone, ZD1839 (an EGFR TKI) alone, or a combination of the two on nude mice injected with a human SCCHN cell line [47]. Tumor growth in the combined treatment was significantly inhibited versus control (*p* < 0.001), ZD1839 (*p* = 0.005), or celecoxib alone (*p* < 0.001). Saba et al. then conducted a Phase I study to establish maximum tolerated dose (MTD) for the combination [48]. The MTD of erlotinib in combination with celecoxib at 400 mg BID was 50 mg per day, and the onset of skin rash was the dose-limiting factor. Twelve patients with OPML, dysplasia and CIS were treated for a median duration of 5.38 months. Overall pathologic response rate was 63%. The average time to development of more severe dysplasia or malignancy was 25.4 months. EGFR and p-ERK downregulation in follow-up biopsies correlated with response to treatment. Although early results seemed promising, COX-2 inhibitors have fallen out of favor due to their adverse cardiac profile, impeding further investigation of the combination in head and neck cancer chemoprevention.

### 4.4. Micronutrients

Several natural compounds and micronutrients (green tea extract, curcumin, resveratrol, soybean extracts, pomegranate juice, broccoli sprout extract, vitamin C, vitamin E, and lysophilized black raspberries) have been under investigation for their efficacy in head and neck cancer chemoprevention [49,50,51,52,53,54,55,56]. These compounds contain high levels of polyphenols with anti-oxidant properties, and are postulated to inhibit carcinogenesis through their action on downstream signaling pathways [53,54,57].

Warner et al. evaluated the preclinical efficacy of topical freeze-dried black raspberries (BRB) on the prevention of OPML progression in at-risk hamster cheek pouch (HCP) mucosa [55]. After 12 weeks, SCC multiplicity (−41.3%), tumor incidence (−37.1%), and proliferation rate (−6.9%) were reduced in HCP receiving BRB. Topical BRBs correlated with an increase in RB1 expression in developing oral lesions.

Bauman et al. investigated the chemopreventive potential of sulforaphane (a bioactive metabolite of glucoraphanin, found in broccoli sprout extracts) using in vitro models of non-cancerous and cancerous mucosal epithelial cells and an in vivo model of 4-NQO induced murine oral cancer [58]. In a non-cancerous cell line and in 4 SCCHN cell lines, the investigators observed induction of NRF2 and its target genes NQO1 and GCLC, which mediate carcinogen detoxication. Sulforaphane also brought about inactivation of pSTAT3, a factor in SCCHN oncogenesis, and significantly decreased the growth of 4-NQO-induced tongue tumors in mice. These findings support further clinical investigation of sulforaphane in the chemoprevention of SCCHN.

Katiyar and co-workers at the University of Alabama reported growth inhibitory effects of phytochemicals on human SCCHN cell lines [59]. Treatment of human SCCHN cell lines with grape seed proanthocyanidins (GSP) significantly decreased cell viability, induced apoptosis and G1 arrest, inhibited expression of Cyclin D1/D2, and cyclin dependent kinases (CDK), downregulated E2F transcription factor, activated caspase-3, and reduced EGFR expression [60]. There was also decreased cell invasion and activation of NF-κB/p65, a downstream target of EGFR, and inhibition of epithelial to mesenchymal transition, a key process involved in disease progression [61]. The same group also tested SCCHN cell lines with honokiol, a phytochemical from the magnolia plant, and observed the inhibition of cell viability, decreased EGFR expression, and EGFR-dependent signaling [62]. When administered to nude mice, similar inhibition of the EGFR signaling pathway was noted. Molecular docking analysis demonstrated that honokiol has stronger binding with EGFR when compared to gefitinib. Taking together these findings and the presumption that micronutrients will cause fewer systemic toxicities, these agents merit further investigation in humans.

Table 1 and Table 2 list the completed chemoprevention trials to date in patients with OPML, and a curatively treated malignancy index, respectively. Table 3 lists studies currently underway.

## 5. Novel Candidate Targets and Endpoints

Multiple international research teams have performed next-generation sequencing of invasive SCCHN [74,75,76,77], of which the TCGA data provides the most comprehensive analysis. A wealth of data has emerged from these efforts, enabling therapeutic trials to be designed that target actionable alterations. This analysis also highlights the differences in molecular profiles of SCCHN with viral versus non-viral etiology, and from varied geographic regions.

Similar information would be invaluable, but is lacking in oral precancerous lesions. Campbell et al. recently made a call for the development of a Pre-Cancer Genome Atlas (PCGA) to capture serial changes in the molecular profiles of premalignant lesions as they progress towards, or regress from malignancy in multiple tumor types [78]. This information would not only serve to develop novel targeted strategies to delay or reverse carcinogenesis, but would also enable the development of prognostic and predictive biomarkers that could serve as tools for early detection, risk stratification, patient selection for studies, and as surrogate endpoints in clinical trials. 

### 5.1. Notch-1

The Notch pathway is intricately involved in several cellular functions, including maintenance of stem cells, proliferation and apoptosis [79]. Exome sequencing has shown Notch-1 to be the second most commonly mutated gene in SCCHN after TP53 [74,77]. Notch signaling can exert either activating or tumor suppressive effects in a variety of solid and hematological malignancies [80]. A bimodal pattern of Notch pathway alterations in SCCHN has previously been reported, with a smaller subset of patients with inactivating Notch-1 receptor mutations and a larger subset exhibiting increased expression or gene copy number [81]. The potential implication of a concerted effort of genomic characterization of OPML was recently demonstrated by the identification of Notch-1 mutations in 54% of primary oral SCC and 60% of premalignant lesions collected from patients in China [82]. In contrast to Notch-1 inactivating mutations previously reported in Caucasian-predominant populations, gain-of-function or activating mutations were predominantly seen in the Chinese sample, signifying a targetable driver in OPML. Although risk of progression to malignancy associated with a Notch-1 mutation is currently unknown, this should be investigated in a larger sample with longitudinal follow-up. Currently, Notch pathway inhibitors are being tested in clinical trials for the treatment of other advanced malignancies. They may have application in the subset of OPML or SCCHN patients with activating mutations as a prevention measure. However, information from an etiologically and ethnically diverse sample of precancerous lesions would be vital to assess the frequency of a driver mutation and enable appropriate patient selection onto pilot studies.

### 5.2. Stat-3

Signal transducer and activator of transcription 3 (Stat-3) relays intra- and extracellular signals to the nucleus, mediates transcription of target genes and thus plays a key role in cell survival, motility and tumorigenesis across several human malignancies [83]. Stat-3 is a constitutively activated oncogenic transcription factor in SCCHN [84]. Its activation is an early event in SCCHN carcinogenesis, implicating that its inhibition may be a potential chemopreventive strategy [85]. Peyser et al. recently demonstrated that targeting of Stat-3 with a small molecule inhibitor, Stattic, exerted a chemopreventive effect against chemically-induced oral cancer in a preclinical mouse model (*p* = 0.04) [86]. Stat-3 inhibitors are currently in development and testing in early-phase clinical trials for the treatment of advanced solid malignancies. Stat-3 as a chemoprevention target in SCCHN merits further exploration.

### 5.3. CCR7

Chemokine receptor 7 (CCR7) is a transmembrane protein that is expressed by migrating cells, to home in to lymphoid tissues which express its ligands [87]. Upregulation of CCR7 expression in SCCHN is associated with increased tumor cell survival, invasiveness, potential for metastases, and resistance to treatment [88,89], making it an important pathway to elucidate to better understand its contribution to tumorigenesis. Mburu and colleagues employed immunohistochemical staining on a tissue microarray of 47 SCCHN patient tumors to determine if CCR7 upregulation impacted progression-free survival (PFS) and overall survival (OS) [90], and found that it was indeed associated with significantly worse median PFS (*p* < 0.013) and OS (*p* < 0.026). Yang et al. reported that praline-rich tyrosine kinase 2 (Pyk-2) is activated when CCR7 binds with its ligand CCL19 in SCCHN cells [91]. The same group of authors then demonstrated that Pyk-2 acts as an important downstream signaling player of the CCR7 pathway in SCCHN by regulating CCL19 dependent tumor cell migration, metastasis and viability [92]. Monoclonal antibodies targeting chemokine receptors are being tested in clinical trials against a variety of cancer types, particularly lymphomas and leukemias [93]. Preclinical testing in SCCHN/pre-malignant cell lines and mice models is warranted for possible exploration as a chemopreventive option.

### 5.4. TAK1

Transforming growth factor β activated protein kinase 1 (TAK1) was initially identified to be a key regulator of inflammation and cell survival [94] and brings about these effects, in part, via the downstream mitogen-activated protein kinases (MAPKs) and nuclear factor kappa B (NF-κB). Pan and colleagues reported that TAK1 activation is associated with increased CCR7 expression in breast cancer cells [95]. Huang et al. confirmed that the converse is also true—TAK1 inhibition by 5Z-7-Oxozeaenol (5Z-O) was associated with suppression of downstream signaling and CCR7 downregulation in breast cancer cell lines, resulting in decreased tumor growth and lymphatic invasion when tested in vivo in mouse axillary lymph nodes [96]. Singh et al. described that TAK1 promotes cell viability in KRAS dependent colon cancer cells [97,98]. Xenografted tumors in mice were created using KRAS dependent cell lines. Selective inhibition of TAK 1 by intraperitoneal injection of 5Z-7-oxozeaenol resulted in significant regression of tumors within 6 days of treatment. Novel TAK1 inhibitors are currently in development for therapeutic trials in KRAS enriched tumor types such as pancreatic cancer [99]. This ubiquitous signaling pathway merits further study in SCCHN cell lines.

### 5.5. NF-κB

NF-κB is a transcription factor implicated for its role in cancer cell survival and acquisition of chemotherapeutic resistance, including to cisplatin [100,101]. Yan and colleagues have reported that highly metastatic SCCHN cell lines overexpress NF-κB (*p* < 0.01) [102], and their in vitro treatment with selective inhibitors reduces cell invasiveness. In nude mice, the authors also found decreased lymph node and lung metastases. Unfortunately, clinical trials with bortezomib alone or in combination with chemotherapy in SCCHN have shown disappointing response rates [103,104]. Trials studying its potential role in combination with radiation with or without systemic therapy have completed accrual and are yet to be reported (NCT00629226, NCT01445405, NCT00011778, NCT00329589). Nonetheless, this remains an important pathway is most tumor types, including SCCHN, and warrants further exploration in the chemoprevention setting.

### 5.6. TP53/p63

p53 is a tumor suppressor protein which serves as a key regulator of genes associated with cell cycle arrest during times of DNA damaging processes such as inflammation [105]. P53 mutation is an early step in SCCHN carcinogenesis, and also the most frequently detected molecular aberration, having been reported in 50–80% of cases in some series [74,106]. It is also associated with alcohol/tobacco use, and worse clinical prognosis [107,108]. Extensive research over several decades has focused on attempting to restore p53 function via drug therapy, but this has not translated into clinical benefits. 

P63, which is a family member of p53, has been reported to be overexpressed in up to 80% of squamous malignancies [109]. Through its upregulation, Rocco and colleagues showed that it facilitates SCCHN cell survival via p73 suppression, [110] which, much like p53, mediates cell apoptosis. Given the significance of this pathway in SCCHN, efforts need to be targeted early on to prevent OPML progression to frank malignancy.

## 6. HPV-Related Oropharyngeal Carcinoma

HPV-Related Oropharyngeal Carcinoma (HPV-OPC) presents a unique challenge for devising chemoprevention strategies, given the distinct biology of the disease. The HPV-16 strain has emerged as an increasingly frequent etiologic factor for the development of oropharynx cancer (OPC) [11]. HPV viral proteins E6 and E7 promote cell cycle progression and viral DNA replication in mucosal epithelial cells via p53 tumor suppressor protein degradation and retinoblastoma tumor suppressor protein ubiquitination, respectively [111,112]. A cross-sectional study of over 5000 participants using their rinses and testing for HPV DNA by polymerase chain reaction (PCR) estimates that approximately 7% of the adult population harbor active oral HPV infection, with the prevalence being higher among men than women [113]. Analysis of registry data from Wayne State University has also revealed racial disparities, with African-Americans having a significantly lower rate of HPV-OPC versus other races (odds ratio 0.14, 95% CI 0.05–0.37), despite adjusting for tobacco and alcohol use [114]. Another recent report by Zandberg et al. from the University of Maryland similarly shows significantly greater likelihood of HPV-OPC patients to be white, and also notes that there has been a significant increase in HPV-OPC among both white and black patients when comparing data from 1992 to 2007 [115]. In the 1990s, 33% of Caucasian patients had HPV-OPC, and none among black patients. In the 2000s, 17.7% of black patients and 54% of Caucasian patients had HPV-OPC, suggesting a rising trend among all races.

Commercially available vaccines contain the inactive L1 viral capsid protein from different HPV sub-types, eliciting a virus neutralizing antibody response and preventing initial infection with the HPV types in the vaccine [116]. In the double-blind randomized Costa Rica Vaccine trial, Herrero et al. evaluated the efficacy of the bivalent HPV 16/18 vaccine in reducing oral HPV infection four years following vaccination [117]. A total of 7466 women were randomized to receive the HPV16/18 vaccine or hepatitis A vaccine as control. At the four-year study visit, the control group had 15 prevalent HPV16/18 infections while the vaccine group had one, resulting in a vaccine efficacy of 93.3% (VE). The study was limited in its assessment because baseline oral HPV status was not obtained, as it was not initially designed to evaluate VE against oral HPV infections. Given the gaps in our knowledge pertaining to the natural history of oral HPV infection, we also cannot directly infer that the vaccine would prevent OPC. Vaccination cannot be used to treat established infections [118] and according to data from the National Immunization Survey-Teen, 2015, community uptake remains lower than the Tdap and meningococcal vaccines (63% for girls and 50% for boys) [119]. To overcome some of these limitations, therapeutic HPV vaccines, which aim to generate a cell-mediated immune response to HPV oncoproteins E6/E7, are being developed for use in people with prevalent HPV infection and would likely be invaluable as a cancer prevention measure [120]. These are currently under investigation.

## 7. Epstein-Barr Virus-Related Nasopharynx Carcinoma

Human infection with Epstein-Barr virus (EBV) is very common among all populations and has life-long persistence [121]. In 1973, EBV was first detected by in situ hybridization in nasopharynx carcinoma (NPC) tumor cells [122], and is now associated with most cases of NPC, particularly in the high-incidence regions of China and south-east Asia [123,124]. EBV encodes several surface glycoproteins of which gp350 is the most abundant, and has been the most widely studied and deployed of all vaccine immunogens [125]. Several early-phase in-human trials have tested recombinant and vaccinia virus expressing gp350 vaccines, and some were able to induce the production of neutralizing antibodies against EBV [126,127,128,129,130]. Therapeutic vaccines, targeting latent membrane proteins (LMP1/2) and aiming to induce cellular immunity, have also been tested in patients with NPC, with some patients demonstrating T-cell and clinical responses [131,132,133,134,135]. These will be further tested in placebo-controlled trials, and may hold promise in future prevention trials for EBV-NPC.

## 8. Immune Checkpoint Inhibition

The activity of PD-1 inhibition in squamous cell cancer of the head and neck has led to FDA approval of pembrolizumab and nivolumab in platinum-refractory recurrent or metastatic disease [136,137,138]. This has been followed by proposals to study PD-1-directed antibodies in oral premalignant lesions. A trial nearing activation [NCT02882282] will administer four doses of pembrolizumab to patients with oral intra-epithelial neoplasia and the molecular high-risk profile of LOH at 3p14 and/or 9p21, plus at least at one additional chromosomal site (4q, 8p, 11p, 13q, or 17p) for patients with no prior oral cancer, or LOH at 3p14 and/or 9p21 for those with a prior history of invasive oral cancer. Given intravenous administration, the high cost of PD-1 antibodies and the approximately 15% risk of high grade toxicities necessitating immunosuppressive therapy or hospitalization, the ultimate utility of this approach is likely to depend on identifying patients who are not just at high risk for subsequent invasive cancer, but who are at high risk for death from head and neck cancer. 

## 9. Nano-Chemoprevention

The use of nanotechnology-based regimens for cancer prevention was first introduced as a concept by Hasan Mukhtar in 2009 as a way to improve the systemic delivery and bioavailability of promising chemopreventive agents [139]. The authors encapsulated green tea polyphenol epigallocatechin-3-gallate (EGCG) in polylactic acid-polyethylene glycol nanoparticles and found that EGCG exerts its proapoptotic, anti-angiogenic effects with over a 10-fold dose advantage. Sulfikkarali et al. evaluated the chemopreventive efficacy of free naringenin (a naturally occurring plant flavonoid known to have anti-inflammatory and anti-cancer effects [140]) versus naringenin-loaded nanoparticles (NARNP) against 7,12-dimethyl benz(a)anthracene (DMBA) induced oral SCC developed in the buccal pouch of golden Syrian hamsters [141]. Oral administration of NARNP completely prevented tumor development in DMBA painted animals whereas 30% of animals treated with free naringenin and DMBA developed oral SCC. Resveratrol is a dietary polyphenol with demonstrated anti-tumor proliferation effect in several tumor models [142]. However, its clinical applicability has been limited due to its extreme photosensitivity, low chemical stability and limited bioavailability. Recently, nanoformulations have been successfully developed to deliver sustained doses of reserveratrol in cell cultures and animal models. Most recently, Li et al. reported significantly greater inhibition of SCCHN cell lines with nanoformulations of salvianolic acid B, compared to an equivalent amount of free salvianolic acid B [143]. Nano-chemoprevention could thus hold promise in overcoming pharmacokinetic, pharmacodynamic and toxicity limitations in a future generation of chemoprevention trials studying promising agents with restricted clinical application on account of these issues. 

## 10. Future Perspectives

Head and neck cancer chemoprevention has entered a molecularly defined era of personalization. However, from our past attempts, we have learned that our greatest payoff would come from improving on our understanding of the biology of the earliest stages of carcinogenesis. Key to this would be a large-scale, multi-institutional effort to genomically characterize precancerous lesions, akin to the TCGA. Given that only a fraction of premalignant lesions progress to invasive cancers, it is important to develop prognostic biomarkers that identify high-risk lesions and predictive biomarkers that can ideally be incorporated into the eligibility criteria, used as targets for novel therapies, and serve as endpoints on the future generation of clinical trials. This strategy would embody the advent of precision medicine in cancer chemoprevention, ensure appropriate utilization of our current knowledge and resources, and maximize our chances at success.

## Figures and Tables

**Table 1:** Randomized controlled squamous cell cancers of the head and neck (SCCHN) chemoprevention trials for patients with oral premalignant lesions (OPML).

Study Author, Year	Intervention	*N*	Endpoint & Results
Hong, 1986 [28]	13-cisretinoic acid (13-CRA) (1–2 mg/kg/d) or placebo × 3 months	44	OPML clinical and histologic response. 13-CRA decreased OPML size and reversed dysplasia
Lippman, 1993 [63]	13-CRA (1.5 mg/kg/d × 3 months), then randomize: 13-CRA (0.5 mg/kg/d × 9 months) or β-carotene (30 mg/d × 9 months)	70	OPML clinical & histologic response. Following high-dose 13-CRA, low-dose 13-CRA better than β-carotene in maintaining response. On long term f/u, no difference in OCFS between arms.
Sankaranarayanan, 1997 [64]	Vit A (3000 IU/week × 12 months) or β-carotene (360 mg/week × 12 months)	160	Complete regression of OPML. Both regimens better than placebo at inducing OPML remission.
Mulshine, 2004 [38]	Ketorolac oral rinse (10 mL of 0.1% sol, swish/spit BID for 30 s × 90 d) or placebo	57	OPML clinical response rate. Negative study
Papadimitrakopoulou, 2008 [65]	Celecoxib 100 mg BID or 200 mg BID or placebo × 12 weeks	49	OPML clinical response rate. Negative study
Papadimitrakopoulou, 2009 [66]	13-CRA (0.5 mg/kg/d × 1 year, then 0.25 mg/kg/d × 2 years) or β-carotene 50 mg/d + retinyl palmitate 25,000 U/d or retinyl palmitate	162	3 month OPML clinical response. Negative study. 3 month OPML response did not correlate with OCFS
Tsao, 2009 [67]	Green tea extract (500, 750 or 1000 mg/m^2^ TID) or placebo × 12 weeks	41	3 month OPML clinical response. Negative study
Armstrong, 2013 [68]	BBIC (swish & swallow BID) or placebo × 6 months	132	OPML clinical response rate. Negative study
Nagao, 2015 [69]	Β-carotene 10 mg daily + Vit C 500 mg daily or placebo × 12 months	46	OPML remission. Negative study
William, 2015 [24]	Erlotinib 150 mg daily or placebo × 12 months	395	OCFS. Negative study. Prospectively validated high-risk LOH

Abbreviations used: 13-CRA (13-Cis-retinoic acid), d (day), Vit (vitamin), sol (solution), BID (twice daily), TID (thrice daily), BBIC (Bowman-Birk Inhibitor concentrate), f/u (follow-up), OCFS (Oral cancer-free survival).

**Table 2:** Randomized controlled chemoprevention trials of SCCHN second primary tumors (SPTs).

Study Author, Year	Intervention	*N*	Results
Hong, 1990 [29]	13-CRA (50–100 mg/m^2^/d) or placebo × 12 months	103	Effective in preventing SPT
Bolla, 1994 [70]	Etretinate (50 mg/d × 1 month, then 25 mg/d) vs. placebo × 24 months	316	No differences in local, regional or distant recurrence.
Jyothirmayi, 1996 [71]	Retinyl palmitate (200,000 IU/week) or placebo × 1 year	106	Higher frequency of recurrences but no SPT in Vit A group
Van Zandwijk, EUROSCAN, 2000 [72]	Retinyl palmitate (300,000 IU/d × 1 year then 150,000 IU/d × 1 year) or *N*-acetylcysteine (600 mg/d × 2 years) or both or neither	2592	No benefit in OS, EFS, or rate of SPT formation with 2 years supplementation
Mayne, 2001 [73]	Β-carotene 50 mg/d or placebo	264	No decrease or delay in SPT
Khuri, 2006 [31]	13-CRA (30 mg/d) or placebo × 3 years	1190	No decrease in rate of SPT or death

Abbreviations used: OS (overall survival), EFS (Event-free survival), d (day).

**Table 3:** Trials currently accruing patients for SCCHN chemoprevention.

NCT #	Population	Intervention	*N*	Phase	Endpoints
NCT02608736	Patients cured of index SCCHN	Valproic acid 1500 mg/d × 3 months vs. placebo	30	0	Change in saliva protein/histone acetylation
NCT01414426	Patients with OPML	Vandetanib vs. placebo daily × 6 months	54	2	Effect on microvessel density
NCT00099021	Patients with OPML	Pioglitazone daily × 12 weeks	21	2a	Reversal of hyperplastic/dysplastic leukoplakia
NCT01504932	Patients with surgically treated oral cancer	LBR lozenges QID × 6 months vs. observation	44	Pilot	Prevention of recurrent oral cancer
NCT02007200	Patients with stages I-IV SCCHN undergoing surgery	Soy isoflavones × 14 days prior to surgery	44	2	Change in p16 methylation & expression of p16, COX-2, VEGF, EGFR, IL6, p53 and BclxL in tumor and non-tumor adjacent mucosa

Abbreviations used: LBR: lyophilized black raspberry, d (day).
